# Genetic deletion of *Rnd3* in neural stem cells promotes proliferation via upregulation of Notch signaling

**DOI:** 10.18632/oncotarget.20247

**Published:** 2017-08-14

**Authors:** Huimin Dong, Xi Lin, Yuntao Li, Ronghua Hu, Yang Xu, Xiaojie Guo, Qiong La, Shun Wang, Congcong Fang, Junli Guo, Qi Li, Shanping Mao, Baohui Liu

**Affiliations:** ^1^ Department of Neurology, Renmin Hospital of Wuhan University, Wuhan, Hubei 430060, China; ^2^ Department of Cell Biology, Hubei Key Laboratory of Cell Homeostasis, College of Life Sciences, Wuhan University, Wuhan, Hubei 430072, China; ^3^ Center for Translational Cancer Research, Institute of Biosciences and Technology, Texas A&M University Health Science Center, Houston, TX 77030, USA; ^4^ Department of Neurosurgery, Renmin Hospital of Wuhan University, Wuhan, Hubei 430060, China; ^5^ Department of Neurosurgery, Huzhou Central Hospital, Huzhou, Zhejiang 313013, China; ^6^ Department of Intensive Medicine, Hubei Cancer Hospital, Wuhan, Hubei 430079, China; ^7^ Cardiovascular Disease and Research Institute of The First Affiliated Hospital, Key Laboratory of Tropical Diseases and Translational Medicine of Ministry of Education, Hainan Medical University, Haikou, Hainan 571199, China; ^8^ Hainan Provincial Key Laboratory for Human Reproductive Medicine and Genetic Research, The First Affiliated Hospital of Hainan Medical University, Haikou, Hainan 570102, China

**Keywords:** *Rnd3*, neural stem cells, Notch signaling, proliferation, stem cells

## Abstract

Rnd3, a Rho GTPase, is involved in the inhibition of actin cytoskeleton dynamics through the Rho kinase-dependent signaling pathway. We previously demonstrated that mice with genetic deletion of *Rnd3* developed a markedly larger brain compared with wild-type mice. Here, we demonstrate that *Rnd3* knockout mice developed an enlarged subventricular zone, and we identify a novel role for Rnd3 as an inhibitor of Notch signaling in neural stem cells. *Rnd3* deficiency, both *in vivo* and *in vitro*, resulted in increased levels of Notch intracellular domain protein. This led to enhanced Notch signaling and promotion of aberrant neural stem cell growth, thereby resulting in a larger subventricular zone and a markedly larger brain. Inhibition of Notch activity abrogated this aberrant neural stem cell growth.

## INTRODUCTION

Neural stem cells (NSCs) exist in the adult central nervous system (CNS) and are involved in the development and progression of various pathological human conditions, such as degenerative diseases and brain injuries. In the adult brain, the subventricular zone (SVZ) of the lateral ventricle and the dentate gyrus of the hippocampus are niches that maintain populations of NSCs and neural progenitor cells [1–3]. The size of the NSCs population in the SVZ at any time is the result of a balance between several ongoing processes, including self-renewal, cell differentiation, and cell death.

The mechanisms regulating neuronal stem cells (NSCs) populations are very complicated, and crucial roles are played by many signaling pathways in regulating NSCs proliferation, and include Notch1 [4], BMP [5], Shh [6], EGFR [4, 7] and Wnt signaling pathways [6].

Components of the Notch signaling pathway are expressed in neuroproliferative regions of the postnatal brain [7], and Notch signaling is an essential regulator of NSCs maintenance and self-renewal during development. Notch signaling may also augment the expansion and differentiation of adult NSCs following stroke, and activated Notch1 (NICD) and its downstream transcriptional targets, including *Hes1*, which activate NSCs proliferation, have been identified in subventricular zone (SVZ) cells following ischemic injury [8–11].

Rho Family GTPase 3 (Rnd3), also known as RhoE, is a small (27 kDa) G protein of the guanosine-5'-triphosphate (GTP) hydrolase family that was discovered using the yeast two-hybrid method by Foster in 1996 [12]. Rnd3 is a monomeric G protein, and contains only one polypeptide chain, which exists only in the GTP-bound form, but not in the guanosine 5'-diphosphate -bound form [13]. Traditionally, the biological function of Rnd3 was thought to be restricted to participation in the formation of actin. However, a recent study found that Rnd3 can regulate other biological processes including cell migration and apoptosis, by inhibiting Rho protein kinase activity [14].

Recently, it has been revealed that Rnd3 played an important role in the nervous system [15, 16]. These studies showed that lack of Rnd3 led to changes in neuronal polarity, and that rats with low Rnd3 expression exhibited neuromotor disturbances and neuromuscular changes, and decreased the number of dendrites and total dendritic length.

In this study, we provided evidence that deletion of *Rnd3* promoted NSCs proliferation by activating Notch1 signaling. *Rnd3* deficiency, both *in vivo* and *in vitro*, resulted in a decrease in Notch intracellular domain (NICD) protein levels, which in turn enhanced Notch signaling activity and promoted aberrant growth of NSCs, thereby resulting in a larger SVZ and a markedly larger brain.

## RESULTS

### Genetic deletion of *Rnd3* resulted in a larger SVZ and more NSCs

**Figure 1:**
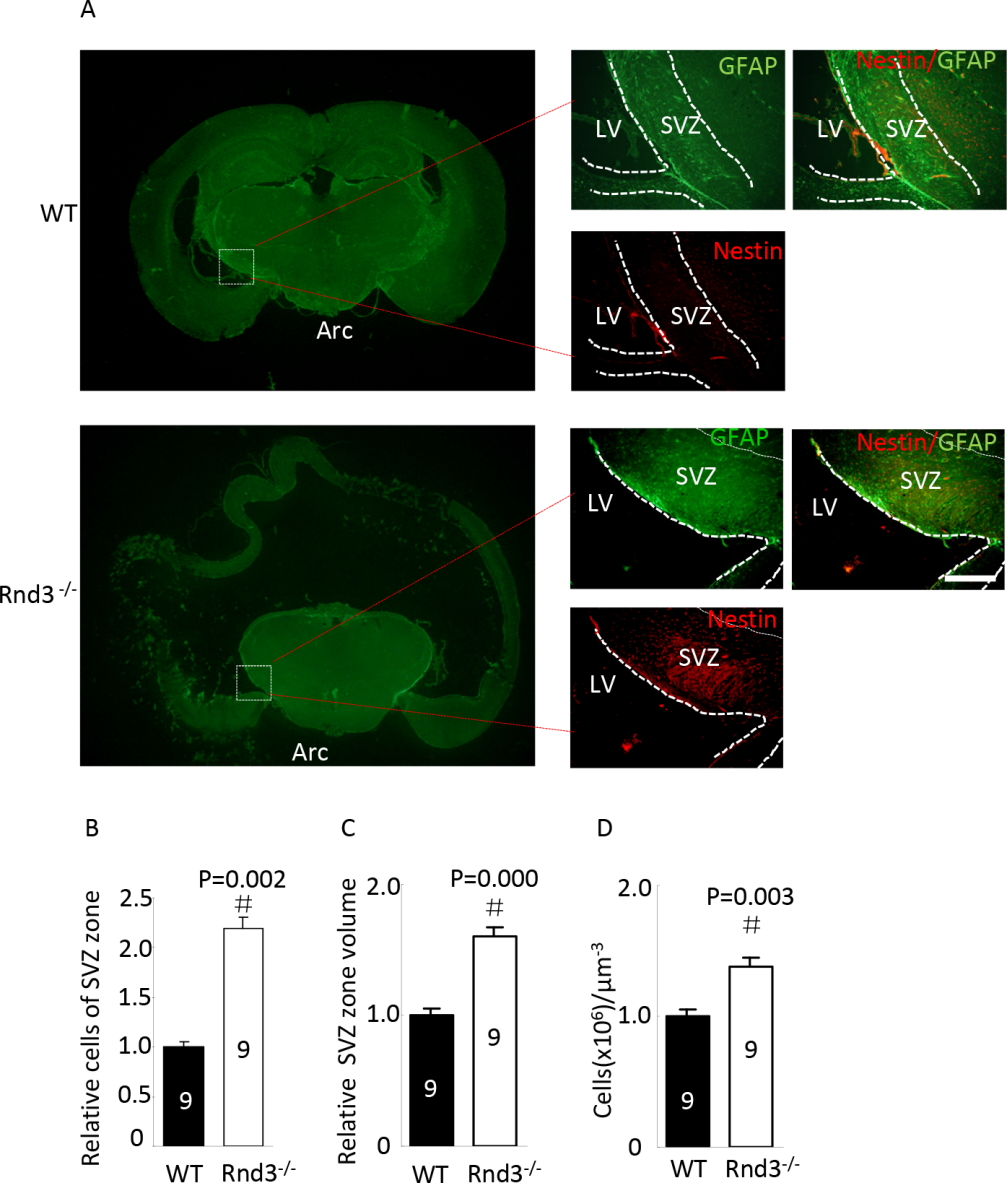
Neural stem cells (NSCs) were universally distributed and enriched in the subventricular zone (SVZ) when *Rnd3* was knocked out **(A)** Double immunofluorescence labeling revealed Nestin and GFAP expression in the SVZ (scale bar, 100 μm). Relative NSCs numbers **(B)** and SVZ volumes **(C)** were increased in *Rnd3*^*-/-*^ mice (n = 9). **(D)** The relative concentration of NSCs in the SVZ was increased in *Rnd3*^*-/-*^ mice (n = 9). Data are presented as means ± SEM, significance was assessed using Student’s t-test, (^#^*P* < 0.05). LV: lateral ventricle, WT: wild-type mouse, Rnd3^-/-^: *Rnd3* knockout mouse.

*Rnd3*^*-/-*^ mice displayed a larger SVZ than the wild type (Figure 1A green) and quantitative estimation indicated that the total volume of the SVZ was significantly larger after *Rnd3* knockout compared with the wild-type mice (*P*<0.05) (Figure 1C). Further examination showed that the SVZ in *Rnd3*^*-/-*^ mice contained a greater density of NSCs (Figure 1A red) and quantitative estimation showed that the overall number and density of NSCs was significantly higher compared with the wild-type mice (*P*<0.05) (Figure 1B, 1D). These observations indicated that a larger NSCs population developed in the *Rnd3*^*-/-*^ mouse SVZ.

### *Rnd3* knockout promoted NSCs proliferation both *in vivo* and *in vitro*, and over-expression of *Rnd3* inhibited NSCs proliferation *in vitro*

**Figure 2:**
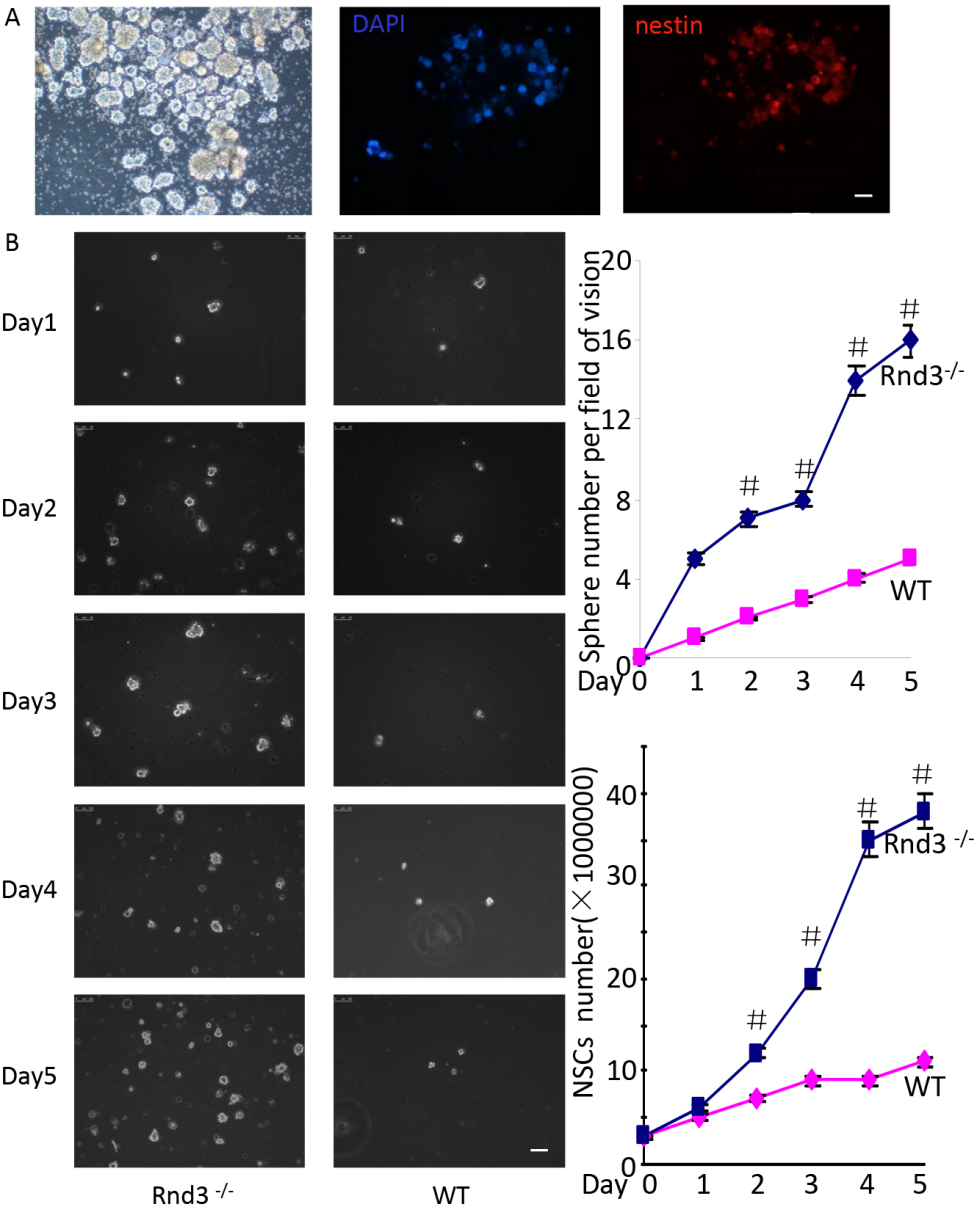
*Rnd3* knockout increased NSCs proliferation **(A)** Neural spheres from wild-type (WT) mice, left: light microscopy images of NSCs spheres, middle-right: double-immunofluorescence labeling revealed Nestin and DAPI expression in NSCs (scale bar, 50 μm). **(B)** Increased neural sphere (left and top right corner) and NSCs numbers (left and lower right corner) were observed in cultures of *Rnd3*^*-/-*^ cells compared with WT cells on days 2, 3, 4 and 5(scale bar, 100 μm). Data are presented as means ± SEM (n = 5) and significance was assessed using Student’s t-test (^#^*P* < 0.05). WT: cells from wild-type mouse SVZ, Rnd3^-/-^: cells from *Rnd3* knockout mouse SVZ.

To investigate the mechanism underlying the enlarged NSCs population and SVZ in *Rnd3*^*-/-*^ mice, NSCs were isolated from the SVZ of 3-day-old mice (wild-type and *Rnd3*^*-/-*^*)* and cultured as described previously [4]. NSCs were then identified by Nestin staining. The cells could form spheres (Figure 2A, left) and were Nestin-positive (Figure 2A, right), indicating that the cells were NSCs.

To assess *Rnd3*-mediated inhibition of NSCs proliferation, equal numbers of cells were cultured in 6-wells plates. Both cell numbers and sphere numbers were significantly higher for the *Rnd3*^*-/-*^ mice (solid blue line) compared with those for the wild-type mice on days 2, 3, 4 and 5 (Figure 2B).

**Figure 3:**
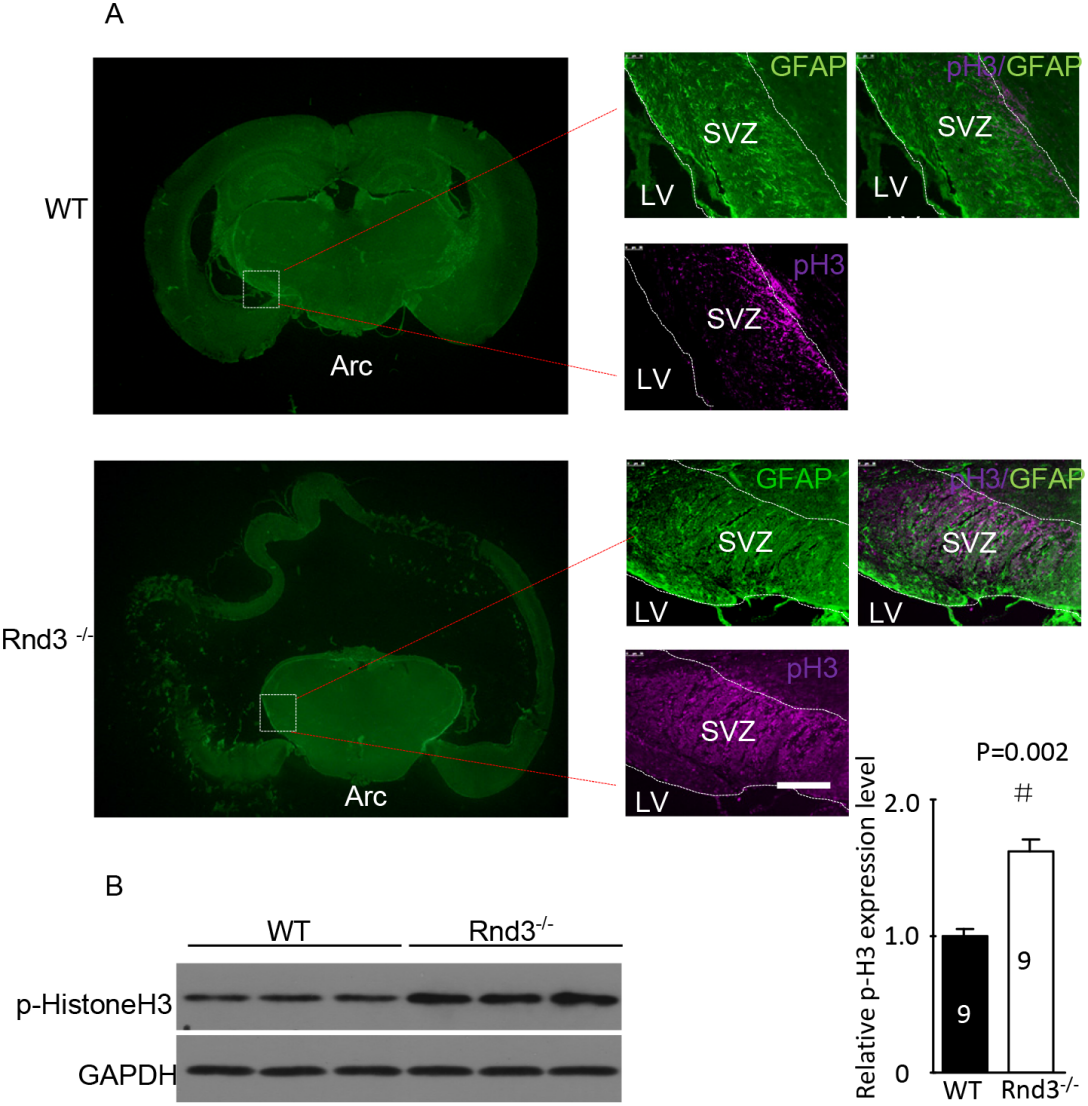
Hyperphosphorylated histone 3 (p-His H3) was enriched in the SVZ when *Rnd3* was knocked out **(A)** Double immunofluorescence labeling revealed GFAP and p-histone H3 expression in the SVZ (scale bar, 100 μm). **(B)** The protein level of the p-His3 was significantly increased in the *Rnd3*^*-/-*^ mouse SVZ. Data are presented as means ± SEM (n = 9); significance was assessed using Student’s t-test, (^#^*P* < 0.05). LV: lateral ventricle, WT: wild-type mouse, *Rnd*3^-/-^: *Rnd3* knockout mouse.

**Figure 4:**
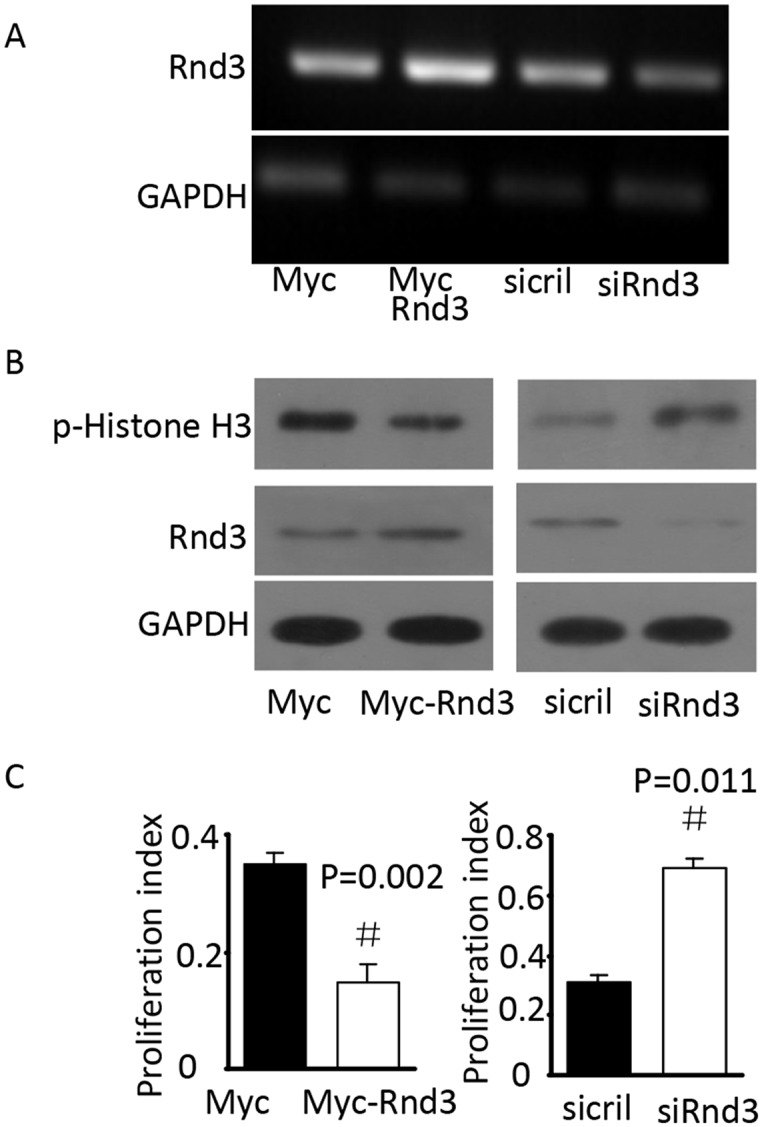
*Rnd3* decreased p-His H3 protein levels *in vitro* **(A)** *Rnd3* mRNA levels in NSCs transfected with *Rnd3* plasmid or SMARTpool siRNA, the bright bands indicated *Rnd3* mRNA expression. **(B)** Rnd3 and p-histone H3 protein levels in NSCs transfected with *Rnd3* plasmid or SMARTpool siRNA. **(C)** Proliferation index of NSCs transfected with Rnd3 plasmid or SMARTpool siRNA. Data are presented as means ± SEM (n = 9); significance was assessed using Student’s t-test, (^#^*P* < 0.05). Myc-Rnd3: Myc-Rnd3 plasmid-transfected NSCs overexpressing *Rnd3*, Myc: control of the Myc-Rnd3 group by transfection of the Myc plasmid, siRND3: downregulation of *Rnd3* by transfection of NSCs with SMART pool siRnd3, siCtrl: control of the siRnd3 group by transfection of SMART pool siCtrl.

To investigate the mechanism underlying the increased number of NSCs in the SVZ of *Rnd3*^*-/-*^ mice, p-Histone H3, which reflects the proliferation of NSCs, was detected in the SVZ by immunofluorescence and western blot analysis. The expression of p-Histone H3 was significantly increased in *Rnd3*^*-/-*^ mice compared with that in wild-type mice (Figure 3A, 3B). Furthermore, quantitative estimation indicated that the expression of p-Histone H3 in the *Rnd3*^*-/-*^ mice was significantly increased compared with that in the wild-type mice (*P*<0.05) (Figure 3C). To confirm this data, *Rnd3* was over-expressed or knocked down in NSCs through the transfection of plasmids over-expressing *Rnd3* or *Rnd3* siRNA, respectively. RT-PCR and western blot indicated that transfection was successful (Figure 4A, 4B), and the expression of p-Histone H3 was significantly downregulated in NSCs overexpressing *Rnd3*, and significantly upregulated in *Rnd3*-knockdown NSCs. Moreover, MTT assays showed that when *Rnd3* was downregulated, the NSCs proliferation rate increased significantly compared with the control group (*P*<0.05), and *vice versa* when *Rnd3* was upregulated (Figure 4C). In summary, these results indicate that overexpressing *Rnd3* can inhibit the proliferation of neuronal stem cells, however, deleting *Rnd3* promotes NSCs proliferation.

### Knocking out *Rnd3* activated NSCs proliferation by promoting Notch1 signaling

**Figure 5:**
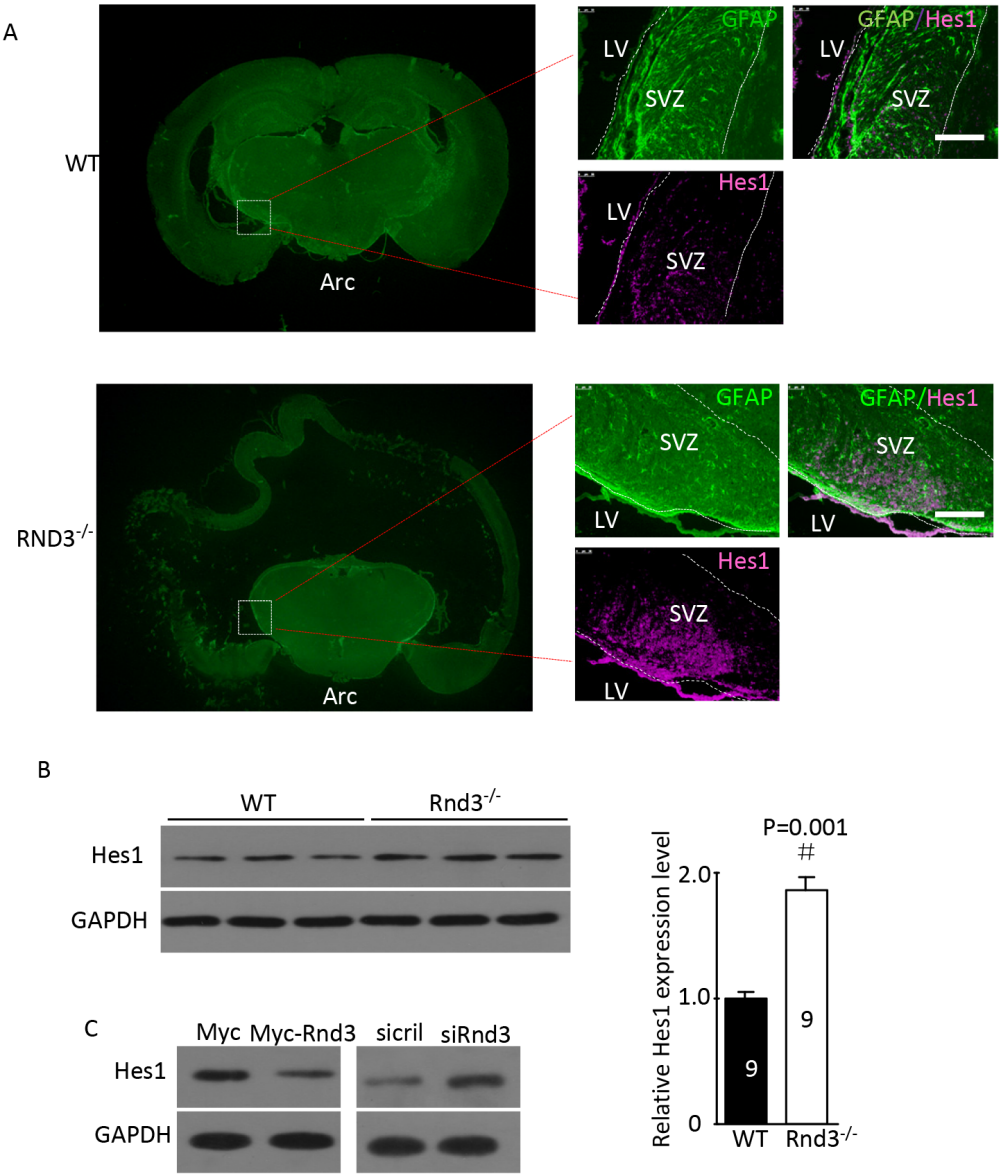
*Rnd3* knockout activated Notch signaling, and *Rnd3* overexpression inhibited Notch signaling **(A)** Double immunofluorescence labeling revealed GFAP and hairy and enhancer of split-1 (Hes1) expression in the SVZ (scale bar, 100 μm). **(B)** *Hes1*, a target gene of Notch signaling, was significantly upregulated in *Rnd3*^*-/-*^ mice compared with wild-type mice. **(C)** Downregulation of *Rnd3* increased Hes1 protein levels in NSCs and *vice versa*. The number on the top of each band represents the average densitometry value from three repeated experiments normalized to GAPDH. Data are presented as means ± SEM (n = 9) and significance was assessed using Student’s t-test (^#^*P* < 0.05). WT: wild-type mouse, Rnd3^-/-^: *Rnd3* knockout mouse.

**Figure 6:**
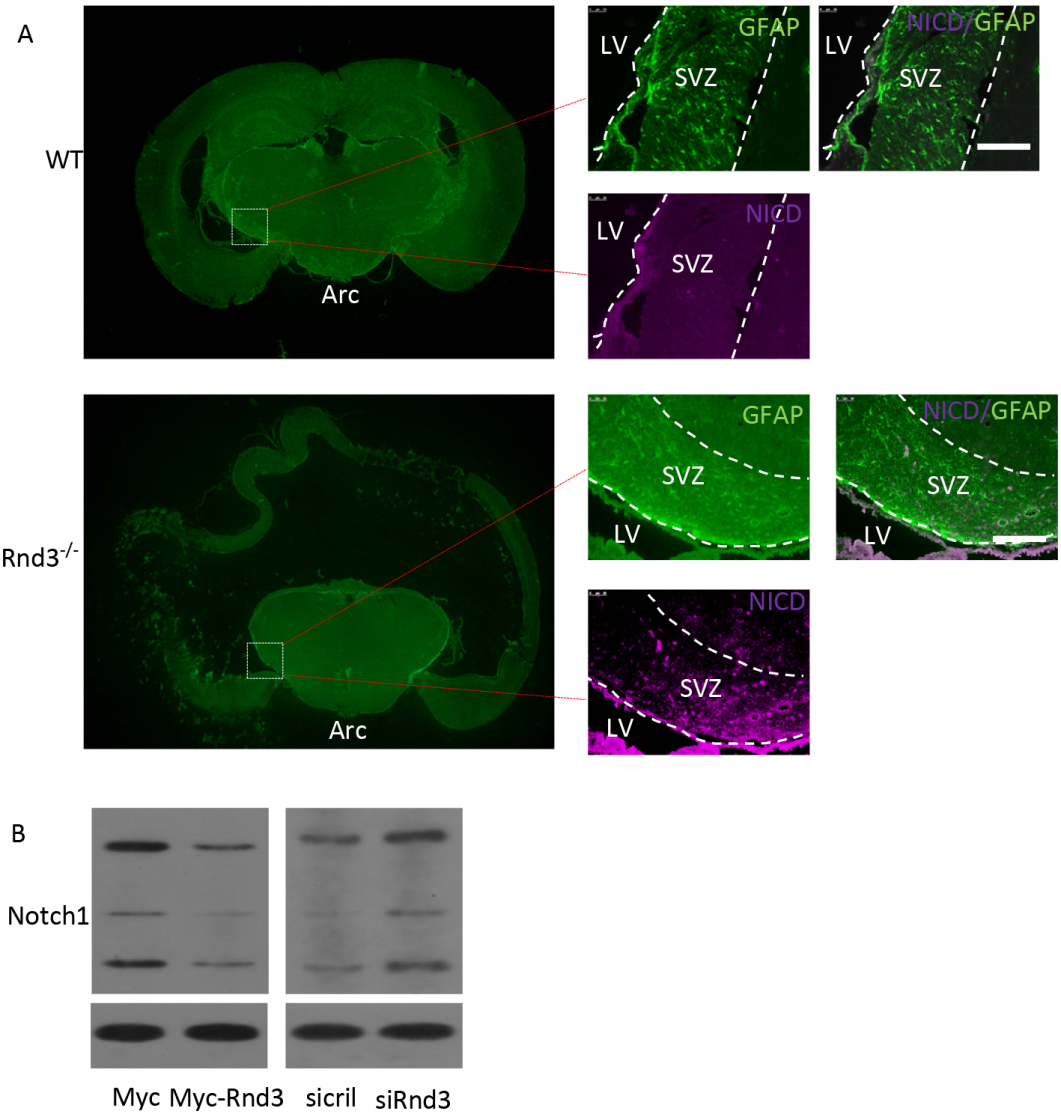
*Rnd3* inhibited Notch signaling by decreasing Notch intracellular domain (NICD) expression levels **(A)** Double immunofluorescence labeling revealed GFAP and NICD expression in the SVZ (scale bar, 100 μm). **(B)** Immunoblot analyses showed that downregulation of *Rnd3* increased protein levels of NICD, the active form of Notch1, in NSCs and *vice versa*. LV: lateral ventricle, WT: wild-type mouse, Rnd3^-/-^: *Rnd3* knockout mouse.

**Figure 7:**
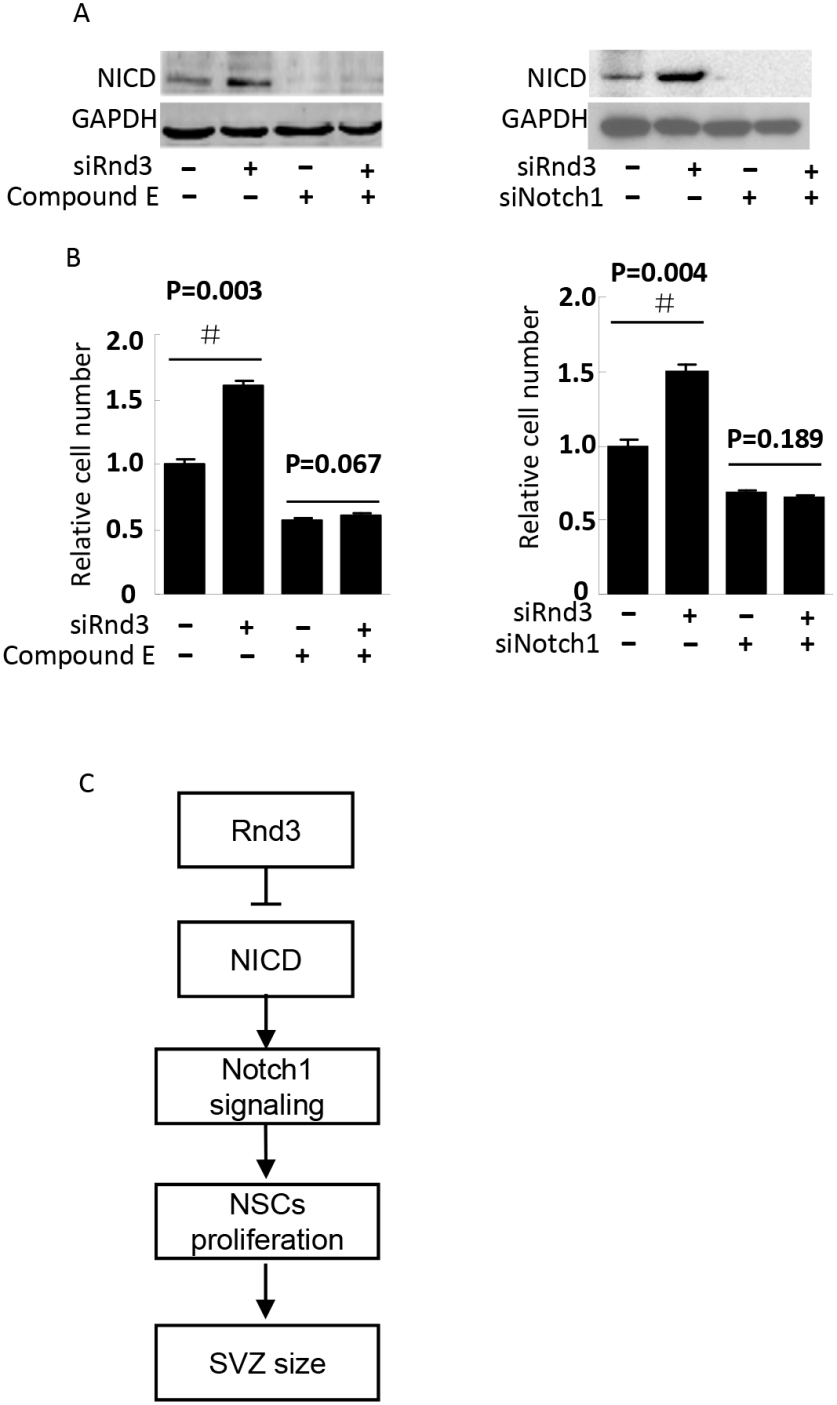
NICD knockdown diminished *Rnd3* deficiency-induced NSCs proliferation NSCs proliferation was assessed by NSCs number, (**A**, left), NICD protein levels when NSCs were treated with Rnd3 SMARTpool siRNA (siRnd3) and compound E, (A, right) NICD protein levels when Rnd3 and Notch1 SMARTpool siRNA (siRnd3 and siNotch1) were transfected into NSCs. (**B**, left) The same result was achieved when Notch1 signaling was blocked by compound E (n = 3), (B, right) *Rnd3* deficiency promoted NSCs proliferation. This enhancement of cell proliferation was completely blocked by NICD knockdown (n = 3). **(C)** A proposed model of *Rnd3* regulation of NSCs proliferation. Data are presented as means ± SEM and significance was assessed using Student’s t-test (^#^*P* < 0.05). siRND3: down-regulation of *Rnd3* in NSCs by transfection of SMART pool siRnd3, siNotch1: down-regulation of Notch1 in NSCs by transfection of SMART pool siNotch1, compound E: a Notch signaling inhibitor.

To detect the mechanism of *Rnd3* deficiency-induced NSCs hyperplasia, *Hes1* expression level was assessed both *in vivo* and *in vitro* (Figure 5A, 5B). *Hes1* is a Notch signaling target gene that functions as a p21 repressor and thus promotes cell proliferation [17]. Our data showed that the Hes1 expression level was significantly increased in the *Rnd3*^*-/-*^ mouse SVZ (Figure 5A, 5B). This was confirmed *in vitro* by western blotting of NSCs, Hes1 levels were clearly decreased when *Rnd3* was over-expressed, while, *Rnd3* knockdown up-regulated Hes1 levels (Figure 5C). Specific immunostaining and western blotting for NICD, the active Notch domain, produced a strong signal in the *Rnd3*^*-/-*^ mouse SVZ (Figure 6A), this was supported by the finding that over-expression of *Rnd3* reduced NICD levels, and downregulation of *Rnd3* increased NICD levels in NSCs *in vitro* (Figure 6B). To examine the relationship between *Rnd3* and activation of Notch signaling, loss- and gain-of-function studies of NSCs were conducted *in vitro*. *Rnd3* knockdown resulted in elevated NSCs numbers, and the effect of *Rnd3* knockdown was abrogated when Notch1 signaling was inhibited either by siRNA specific for Notch1, or by compound E, a Notch signaling inhibitor (Figure 7).

## DISCUSSION

NSCs, which are present both during development and progression in adults, are most commonly defined by their ability to self-renew and their capacity to generate all of the major cell types in the CNS including oligodendrocytes, astrocytes, and neurons.

In the adult brain, the SVZ of the lateral ventricle and the dentate gyrus of the hippocampus are niches that maintain populations of NSCs and neural progenitor cells. One study reported that cell proliferation appears to be decreased in the subgranular zone and SVZ of patients with Parkinson’s disease [18]. Endogenous neurogenesis in the adult mammalian brain occurs to repair or replace lost neurons.

Rnd3 is an atypical member of the Rho GTPase family in that it lacks detectable GTPase activity. The best characterized functions of Rnd3 are its inhibitory effect on Rho kinase-mediated biological functions, including actin cytoskeleton formation, phosphorylation of myosin light chain phosphatase, and apoptosis [19–21]. Two recent mouse studies revealed an indispensable role of Rnd3 in mouse neuron development [15, 22], and we have reported that Rnd3 regulated 293T and glioblastoma cell proliferation both *in vitro* and *in vivo* through Notch1 signaling. Here, we found that Rnd3 can regulate NSCs proliferation. This result extends the understanding of the role of Rnd3 in regulating of cell proliferation. NSCs are critical for CNS development and maintenance of function, and inhibition of NSCs proliferation is a major cause of degenerative disease. Therefore, the regulation of Rnd3 in NSCs proliferation may provide a new therapeutic target in these diseases.

Notch signaling has been shown to regulate a broad range of events during embryonic and post-natal development, including proliferation, apoptosis, border formation and cell fate determination. The mechanism of Rnd3-mediated Notch regulation was first revealed in our recent Rnd3-knockout mouse study [23]. We demonstrated that Rnd3 physically interacts with NICD, the active Notch domain, and regulates NICD availability by mediating NICD protein ubiquitination. Rnd3 deficiency inhibits this process and results in an increase in NICD levels in ependymal cells, thereby augmenting Notch signaling [23]. In the present study, we confirmed the inhibitory regulation of Notch1 by Rnd3 in NSCs. Interestingly, the opposite regulatory relationship between Rnd3 and Notch1 signaling has been reported recently in squamous cell carcinoma, in a study that showed that *Rnd3* was a transcriptional target of Notch1, and that Rnd3 promoted Notch signaling by facilitating NICD nuclear translocation through importin β1 in skin epithelial cancer cells [24]. These two different functions of Rnd3-medaited Notch regulation may indicate the importance of maintaining Notch signaling integrity; regulation may be positive or negative depending on the cellular context. In tissues in which Notch1 signaling activity is too high, Rnd3 may act as a “brake” on the Notch regulatory complex; conversely, Rnd3 may promote Notch activity at low activity levels. Thus, Rnd3 may act to balance Notch1 signaling activity.

In summary, we demonstrate that genetic deletion of Rnd3 results in NSCs hyperplasia, resulting in a larger SVZ.Moreover, we show thatRnd3 is a novel regulator of Notch. Genetic or siRNA knockdown of Rnd3 attenuates NICD protein degradation, resulting in an increase in Notch signaling activity, which promotes cell proliferation and contributes to increased SVZ size (Figure 7C). The identification of this effect of Rnd3 on NICD adds a new regulatory layer to Notch signaling. Given the fundamental role of the Notch pathway in NSCs signaling and cell-to-cell communication, the present findings indicate a potential new target for degenerative disease.

## MATERIALS AND METHODS

### Generation of *Rnd3* knockout mouse lines

*Rnd3* knockout mice were derived from a gene trap ES cell line generated in the Texas Institute for Genomic Medicine and were kindly provided by Professor Jiang Chang. The target vector was inserted into intron 2 of *Rnd3*, and mutant mice were breed with the C57BL/6 strain, as described previously [25]. The age of all mice used in this study was 3-day-old. All animal experiments were approved by the Institutional Ethics Committee of the Faculty of Medicine at Renmin Hospital of Wuhan University.

### Real time PCR

The primers and procedures used were described in our previous study [16].

### Immunofluorescence and immunoblotting

The antibodies specific for Rnd3, Notch1, cleaved Notch1, hairy and enhancer of split-1 (Hes1), p-His H3, and GAPDH; the SMARTpool siRNA for Rnd3 knockdown; and the Myc-Rnd3 plasmid for Rnd3 overexpression that were used in this study are described in our previous work [16]. Antibodies specific for Nestin (ab6142) and GFAP (ab4674) were both purchased from Abcam.

### Isolation and culture of NSCs, and transient gene transfection

NSCs were isolated from the SVZ of 3-day-old C57BL/6 mice (wild-type and *Rnd3*^*-/-*^) and cultured as described previously [4]. NSCs were then identified with Nestin staining, and plated in 6-well plates (3×10^5^ cells/plate). The numbers of spheres were counted daily under light microscopy observation from day 1 to 5 after plating, and the numbers of NSCs were counted from day 1 to 5 after spheres were disrupted by trypsin in another independent experiment.

All transient gene transfections were conducted using the NEON transfection system (MPK5000; Life Technologies). Images were acquired by fluorescence microscopy.

### Calculation of total SVZ volume

The layer of the SVZ with the largest area was selected and its area was calculated and defined as S. The SVZ layer that appeared first was taken as the first layer, and that which appeared last as the last layer. The total thickness of the SVZ was calculated as the number of layers multiplied by the thickness per layer and defined as H. The total volume of the SVZ was calculated using the following formula: 0.5 × S × H. Nine 3-day-old Rnd3^-/-^ or wild-type mice were selected, and the mean of SVZ volume was calculated.

### MTT assay

Forty-eight hours after transfection with plasmids for Myc, Myc-Rnd3, or siCtrl, or siRnd3 siRNAs, cells were plated in 96 well plates (3×10^4^ cells/plate), and incubated with 5 mg/ml MTT solution for 4 h. The formazan product was solubilized with 150 μL dimethylsulfoxide (DMSO). Cell proliferation was detected by measuring the absorbance at 490 nm with a microplate reader. The proliferation index was calculated as follows: (post-treatment MTT - pre-treatment MTT)/ pre-treatment MTT.

### Brain sectioning

Brain sections were prepared from 3-day-old mice (wild-type and *Rnd3*^*-/-*^), Mice were euthanized in carbon dioxide and then the whole brain was dissected. Brains were immediately fixed in 10 ml 4% paraformaldehyde for 48 hours, and then dehydrated by gentle rocking at room temperature in an ethanol series of 70% ethanol for 2 hours, 95% ethanol for 2 hours twice, and 100% ethanol for 1 hour twice. After dehydration, brains were gently rocked at room temperature in Histone clear1 for 30 min and Histone clear2 for 30 min. Brains were then embedded in paraffin wax 1 (60°C) for an hour, paraffin wax 2 (60°C) overnight and paraffin wax 3 (60°C) for 2 hours. After embedding, 5-μm-thick coronal sections were cut and stained.

### Quantification of NSCs in mouse SVZ

The total brightness of the layer of the SVZ with the largest area was detected using Bandscan software and defined as N. The total number of cells was calculated using the following formula: 0.5 × N × H. The means of cell number across nine 3-day-old Rnd3^-/-^ and wild-type mice were calculated.

### Statistical analysis

Data are expressed as mean ± standard error of the mean. For multiple comparisons, one-way analysis of variance was used followed by the Holm–Sidak test. For two-group comparisons, an unpaired, two-tailed student’s *t*-test was used (SigmaPlot, version 11.0). *P*< 0.05 was considered significant.

## Abbreviations

*Rnd3*: Rho Family GTPase 3
NSCs: neural stem cells
NICD: Notch intracellular domain
Hes1: hairy and enhancer of split-1
SVZ: subventricular zone
WT: wild-type
